# Quantitation of DNA methylation by melt curve analysis

**DOI:** 10.1186/1471-2407-9-123

**Published:** 2009-04-24

**Authors:** Eric Smith, Michael E Jones, Paul A Drew

**Affiliations:** 1 Discipline of Surgery, School of Medicine, The University of Adelaide, Royal Adelaide Hospital, South Australia 5005, Australia; 2 School of Medicine, Flinders University, Bedford Park, South Australia 5042, Australia; 3 School of Nursing and Midwifery, Flinders University, Bedford Park, South Australia 5042, Australia

## Abstract

**Background:**

Methylation of DNA is a common mechanism for silencing genes, and aberrant methylation is increasingly being implicated in many diseases such as cancer. There is a need for robust, inexpensive methods to quantitate methylation across a region containing a number of CpGs. We describe and validate a rapid, in-tube method to quantitate DNA methylation using the melt data obtained following amplification of bisulfite modified DNA in a real-time thermocycler.

**Methods:**

We first describe a mathematical method to normalise the raw fluorescence data generated by heating the amplified bisulfite modified DNA. From this normalised data the temperatures at which melting begins and finishes can be calculated, which reflect the less and more methylated template molecules present respectively. Also the *T50*, the temperature at which half the amplicons are melted, which represents the summative methylation of all the CpGs in the template mixture, can be calculated. These parameters describe the methylation characteristics of the region amplified in the original sample.

**Results:**

For validation we used synthesized oligonucleotides and DNA from fresh cells and formalin fixed paraffin embedded tissue, each with known methylation. Using our quantitation we could distinguish between unmethylated, partially methylated and fully methylated oligonucleotides mixed in varying ratios. There was a linear relationship between *T50 *and the dilution of methylated into unmethylated DNA. We could quantitate the change in methylation over time in cell lines treated with the demethylating drug 5-aza-2'-deoxycytidine, and the differences in methylation associated with complete, clonal or no loss of MGMT expression in formalin fixed paraffin embedded tissues.

**Conclusion:**

We have validated a rapid, simple in-tube method to quantify methylation which is robust and reproducible, utilizes easily designed primers and does not need proprietary algorithms or software. The technique does not depend on any operator manipulation or interpretation of the melt curves, and is suitable for use in any laboratory with a real-time thermocycler. The parameters derived provide an objective description and quantitation of the methylation in a specimen, and can be used to for statistical comparisons of methylation between specimens.

## Background

Methylation of DNA is a common epigenetic change which is important for the normal functioning of the cell. Methylation occurs almost exclusively on cytosines in the setting of a CpG dinucleotide. Most CpGs are methylated in the genome, except for those in the majority of CpG dense regions (CpG islands) found at the 5' end of around 50% of mammalian genes. Abnormal methylation is implicated in a number of disease processes. This applies particularly in cancer where there is genome wide hypomethylation together with hypermethylation of many CpG islands, which can silence tumor suppressor genes. There is great interest in assessing methylation because it may have diagnostic or prognostic value, or be a predictive marker for therapy. It is therefore important to have simple, accurate and inexpensive techniques for measuring methylation, which are suitable for use in a range of laboratories.

There are a number of qualitative and quantitative methods for the analysis of methylation, each with its own advantages and disadvantages [1,2]. Many of these methods rely on treating the DNA with sodium bisulfite. This deaminates unmethylated cytosines to uracils while methylated cytosines remain unchanged, resulting in templates that differ in sequence between the unmethylated and methylated forms. Regions can be amplified by PCR using primers specific for bisulfite modified DNA and, depending on the design of the assay, the methylation status of individual CpGs, or of a region containing a number of CpGs, can then be obtained. Techniques which measure the methylation at each CpG within a target region include bisulfite sequencing [3], pyrosequencing [4], and matrix-assisted laser desorption/ionization time-of-flight mass spectrometry (MALDI-TOF MS) [5]. These techniques are relatively expensive to perform, require multiple steps, are time consuming, or require expensive hardware that is not readily available in many laboratories. The commonly used methylation-specific PCR (MSP) [6] does not suffer from these disadvantages, but the primers are more demanding to design, and the assay only measures methylation of CpGs located near the 3' end of the primers.

For many purposes an estimate of the overall methylation of all CpGs within a region is as useful as knowing the methylation at specific CpGs. We describe an objective assessment of overall methylation using melt curve analysis, a technique first described by Ririe to differentiate between desired and undesired products of a PCR [7]. In melt curve analysis PCR products are slowly heated in the presence of double-stand DNA (dsDNA) specific fluorescent dyes such as SYBR Green I, LCGreen, SYTO9 or EvaGreen. With increasing temperature the dsDNA denatures (melts), releasing the fluorescent dye with a resultant decrease in the fluorescent signal. The temperature at which dsDNA melts is determined by factors such as nucleotide sequence, length and GC/AT ratio. A methylated sequence of DNA, following bisulfite modification, will maintain a higher GC/AT ratio and so melt at a higher temperature than its unmethylated equivalent. Melt curve analysis can detect a single base difference [8]. Worm applied this principle to DNA methylation analysis [9], and a number of variations have since been described, such as methylation-sensitive high resolution melting [10] and dissociation analysis [11]. In each of these methods methylation is assessed visually. In melting curve analysis-methylation (MCA-Meth) the ratio of methylated to unmethylated amplicons was calculated from the respective heights of the derivative peaks (-dF/dT), but this method of quantitation cannot be applied to samples containing partially methylated molecules [12].

We describe a simple mathematical approach to generate normalised melt curves from the raw fluorescence melt data obtained following PCR. We can then calculate from the normalised melt curve the melt temperature (*T50*), the temperature at which 50% of the molecules in the PCR product are melted, which reflects the average methylation of all the CpGs in the region amplified, and the temperatures at which melting begins and is complete, which reflect the heterogeneity of methylation of the alleles within the amplified region. These parameters provide an objective description and quantitation of the homogeneous and heterogeneous methylation in a specimen, and can be used to compare methylation between specimens.

## Methods

### Primers and oligonucleotides

Primers (Table 1), and oligonucleotide sequences representing the bisulfite modified fragments of the CDKN2A promoter (Table 2), were synthesized by GeneWorks (Thebarton, SA, Australia). Primers for melt curve analysis were designed to amplify both methylated and unmethylated bisulfite modified DNA, but not unmodified DNA. Our primer design guidelines were as follows.

**Table 1:** Primers used in this study

Gene	Primer sequences 5'-3'	Location^a^	Size^b^	CpGs^c^
CDKN2A	Forward-GAAGAAAGAGGAGGGGTTGGTTGGTTATT Reverse-ACCTACTCTCCCCCTCTCCGCAA	chr9:21964847 – 21964930	84	6
TIMP3	Forward-GGYGGTATTATTTTTTATAAGGATTTG Reverse-AAACCCCRCCTCRAACTATTAAA	chr22:31527488 – 31527645	158	10
MGMT	Forward-IGIGTTTIGGATATGTTGGGATAGTT Reverse-ACIAAACIACCCAAACACTCACCAAA	chr10:131155461 – 131155570	110	12

Y – C/T

R – A/G

I – inosine

^a^Genome location, as determined by UCSC Genome Browser Database (GBD, http://genome.ucsc.edu) Human March 2006 (hg18) assembly.

^b^Size of the PCR product.

^c^Number of CpGs in the PCR product between the primer binding sites.

**Table 2:** Oligonucleotide sequences representing a fragment of the CDKN2A promoter

Oligonucleotide	Sequence
Unmethylated	gaagaaagaggaggggTtggTtggtTaTTagagggtgggg**TG**gaT**TGTG**tg**TG**Tt**TG**g**TG**gTtgCGgagagggggagagTaggT
Partially methylated	gaagaaagaggaggggTtggTtggtTaTTagagggtgggg**TG**gaT**TGTG**tg**CG**Tt**CG**g**CG**gTtgCGgagagggggagagTaggT
Fully methylated	gaagaaagaggaggggTtggTtggtTaTTagagggtgggg**CG**gaT**CGCG**tg**CG**Tt**CG**g**CG**gTtgCGgagagggggagagTaggT

The oligonucleotide sequences representing a bisulfite modified fragment of the CDKN2A promoter that were either unmethylated, partially methylated, or fully methylated. The sequence to which the primers bind is underlined. The location of the six CpGs between the primer binding sites are in bold. Thymines which are generated following bisulfite modification and amplification are capitalised.

1. They should contain at least one T corresponding to a non-CpG C within the last three nucleotides at the 3'-end of the primer, to increase the likelihood of amplification of only bisulfite modified template.

2. They should not contain CpGs, but when this is unavoidable the number of CpGs are minimised and placed as far as possible toward the 5'-end of the primer. In these cases primers are designed with either an inosine or a degenerate base (C/T in the forward primer, or G/A in the reverse primer) so that templates with both methylated and unmethylated CpGs would be amplified.

3. There should be limited self-complementarity and limited complementary sequences between primer pairs. These parameters can be assessed using freely available software tools including Oligo Calc: Oligonucleotide Properties Calculator to test for self-complementarity http://www.basic.northwestern.edu/biotools/oligocalc.html) and Amplify (version 3.1.4, http://engels.genetics.wisc.edu) to test for potential primer-dimers.

4. They should amplify a product which is approximately 80 – 160 bp in length.

5. The primers should be approximately 20 – 30 bp in length.

6. The primer pairs should have melting temperatures as similar as possible, but differing by no more than 2°C.

### Reference DNA

Bisulfite modified genomic DNA prepared from the lymphocytes of healthy donors was used as unmethylated reference [13]. For the methylated reference, 2 μg of lymphocyte genomic DNA was treated with 10 U of M.SssI CpG Methylase (New England BioLabs Inc., Beverly, MA, USA) for 16 h at 37°C in a 50 μL reaction volume containing 160 μM S-adenosylmethionine and NEBuffer 2 (50 mM NaCl, 10 mM Tris-HCl, 10 mM MgCl_2_, 1 mM dithiothreitol, pH 7.9). Methylase treated DNA was precipitated with 150 μL of 100% ethanol and centrifuged for 15 min at 4°C. The ethanol was removed and the DNA pellet was air-dried under vacuum. The DNA was resuspended in 18 μL of ultra-pure water (UPW, Fisher Biotec Australia, West Perth, WA, Australia), and bisulfite modified.

### Cell culture

The esophageal adenocarcinoma cell line OE33 was cultured in RPMI 1640 supplemented with 10% foetal bovine serum, at 37°C, in air enriched with 5% CO_2_. Triplicate cultures of OE33 were grown for 24 h, then treated with either 1 μM 5-aza-2'-deoxycytidine (aza) (Sigma-Aldrich, Saint Louis, MO, USA) or vehicle (0.0027% v/v final concentration acetic acid) for 48, 72 or 120 h. The cells were incubated for a further 24 h in fresh medium not containing aza or vehicle and harvested. The DNA was isolated using TRIzol (Invitrogen, Carlsbad, CA, USA) according to the manufacturer's instructions.

### Colorectal carcinoma tissue specimens

For each colorectal carcinoma (CRC) tissue, two 10 μm formalin fixed paraffin embedded (FFPE) sections were de-waxed with xylene, washed with 100% ethanol, re-hydrated with UPW and air-dried under vacuum. Sections were then digested in a solution consisting of 20 μL 10 mg/ml proteinase K (Promega, Madison, WI, USA) and 200 μL 100 mM NaCl, 10 mM Tris-HCl, 10 mM EDTA and 0.5% (w/v) sodium dodecyl sulfate for 48 h at 55°C, adding 20 μL fresh 10 mg/ml proteinase K after the first 24 h. Protein was removed by precipitation with 80 μL of 6 M NaCl. The DNA was precipitated with 700 μL of 100% ethanol, washed with 70% ethanol, air-dried under vacuum and resuspended in 100 μL UPW. This study was approved by the Research Ethics Committee of the Royal Adelaide Hospital. The study complied with the appropriate institutional guidelines.

### Bisulfite modification

Genomic DNA (2 μg) was bisulfite modified as previously described [14,15]. Bisulfite modified DNA from lymphocytes and cell lines were resuspended in UPW at a volume of 100 μL, and from FFPE tissue in a volume of 20 μL.

### PCR and melt analysis

Bisulfite modified DNA (1 μL) was amplified using QuantiTect SYBR Green PCR Kit (Qiagen, Hilden, Germany) containing a final concentration of 0.5 μM of each primer (Table 1) in a final reaction volume of 15 μL. The primers and PCR conditions were specific for bisulfite modified DNA, and did not amplify unmodified DNA. The PCR was performed using a Rotor-Gene 3000 (RG3000, Corbett Life Science, Sydney, NSW, Australia) with a 95°C activation step for 15 min; 95°C for 30 s, 55°C for 60 s for 45 cycles; and a final extension step of 72°C for 4 min. The melt of the PCR product was performed from 60 to 90°C, rising in 0.5°C increments, waiting for 30 s at the first step and for 5 s at each step thereafter, and acquiring fluorescence at each temperature increment.

### Statistics

Two groups were compared using the Wilcoxon-Mann-Whitney test, and three groups were compared using the Kruskal-Wallis one-way analysis of variance by ranks [16]. More than three groups were compared with one-way analysis of variance with Tukey multiple comparison post-test. All statistics were considered significant when the two tailed P = 0.05.

## Results

### Algorithm: analysis of melt curves from raw melt data

**Figure 1:**
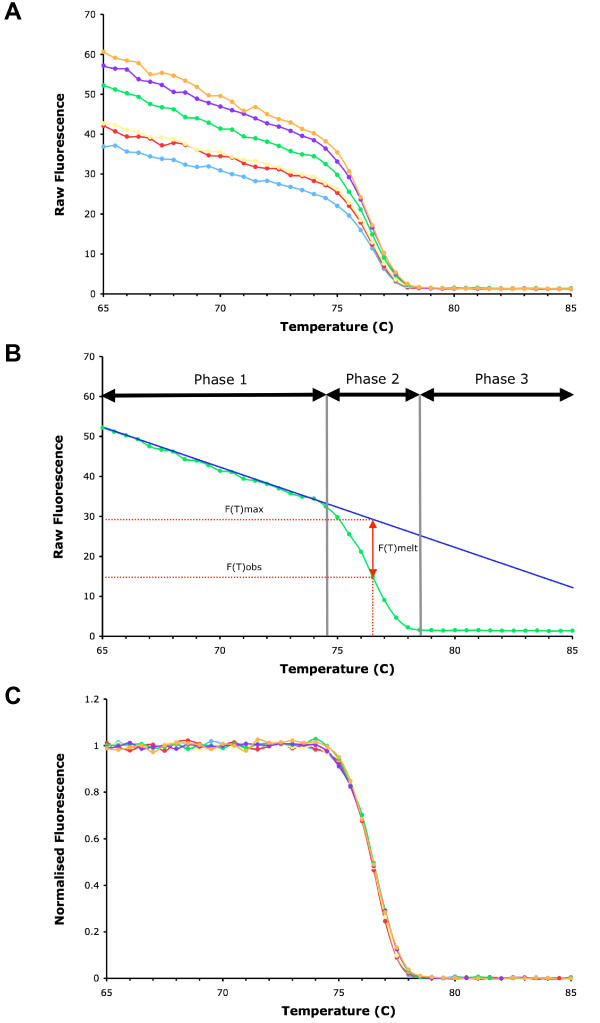
**Raw and normalised melt curves**. A) Typical examples of raw fluorescence melt curves. Six replicates of an oligonucleotide representing a fragment of the unmethylated CDKN2A promoter were amplified using the CDKN2A primers and QuantiTect SYBR Green PCR Kit, then melted by increasing the temperature from 60 to 90°C, rising in 0.5°C increments, waiting for 30 s at the first step and for 5 s at each step thereafter, acquiring fluorescence at each increment. Raw fluorescence was plotted against temperature. The plots show that the magnitude of the starting fluorescence may vary significantly even between replicates. B) Graph of raw fluorescence plotted against temperature from one sample from Figure 1A shows that there are three distinguishable phases. In Phase 1 there is a linear decrease in fluorescence with increasing temperature (represented as a solid blue line). In Phase 2 there is a rapid reduction in fluorescence which is a combination of the linear decrease and the melting of dsDNA. During Phase 2 the observed fluorescence (*F(T)obs*) is less than the fluorescence expected from the Phase 1 alone (*F(T)max*). The reduction in fluorescence due to the melting of the dsDNA alone at any given temperature (vertical dotted red line) is the difference between *F(T)max *and *F(T)obs*, *F(T)melt *(solid red line with arrow heads). In Phase 3 the dsDNA is fully melted and there is no further decline in fluorescence. C) Normalised melt curves for the six replicates in Figure 1A. The normalised fluorescence was calculated by the formula *F(T)obs*/*F(T)max *at every given temperature increment. Normalised fluorescence expressed as a percentage was then plotted against temperature.

A graph of the raw fluorescence plotted against temperature for a typical melt of dsDNA PCR products using the RG3000 is shown in Figure 1A. The magnitude of the starting fluorescence often varies significantly between samples, making direct comparisons difficult. To facilitate comparisons, the raw fluorescence data can be normalised, as described below. Figure 1B shows that the graph of raw melt data for a sample has three distinguishable phases. In Phase 1 there is a linear decrease in fluorescence with increasing temperature. This decrease can be represented mathematically. There is a constant background amount of fluorescence, and added to this is a linearly decreasing amount which can be calculated from the regression line which best fits the fluorescent data over the linear reduction phase (represented as a solid blue line in Figure 1B). In Phase 2, the rapid reduction in fluorescence is a combination of the linear decrease and the melting of dsDNA. During this phase the observed fluorescence (*F(T)obs*) is less than the fluorescence expected from the Phase 1 alone (*F(T)max*). In Phase 3, the final plateau phase, the dsDNA is fully melted and there is no further decline in fluorescence.

At each temperature point it is possible to calculate how much fluorescence is lost as a result of melting of the dsDNA. The amount of fluorescence in the sample at any given temperature, if the dsDNA does not melt and assuming that the reduction in fluorescence is linear with increasing temperature, can be calculated from the extrapolation of the regression line of the Phase 1 decline. The reduction in fluorescence (*F(T)melt*) due to the melting of the dsDNA at any given temperature, is the difference between the fluorescence predicted at that temperature from the regression line (*F(T)max*) and the observed value (*F(T)obs*), or *F(T)melt = F(T)max – F(T)obs*.

The normalised fluorescence at any given temperature is the ratio of the amount of fluorescence observed to the amount of fluorescence predicted if no melting of the dsDNA had occurred (*F(T)obs*/*F(T)max*), expressed as a percentage. Normalised fluorescence values can then plotted against temperature as normalised melt curves (Figure 1C). The initial phase is a flat line, as there is no reduction in fluorescence due to specific dsDNA melting. Reduction in fluorescence due to specific melting of the dsDNA begins at the take-off temperature (*Tto*) and finishes at the touch down temperature (*Ttd*), where the fluorescence reaches background. We calculated these as the 95% and 5% percentile fluorescence respectively, but other values could be easily used.

Normalising the raw melt data facilitates comparisons between melt curves of different samples, and to unmethylated and fully methylated DNA references. Two parameters which describe the methylation of the population of DNA templates for the region amplified can be determined from the normalised curve. The temperature range over which melting occurs, the difference between take-off and touchdown temperatures (*Ttd – Tto*), will be greater if there is a mixture of methylated and unmethylated molecules than if there is a homogeneous population, making this a measure of methylation heterogeneity. The extent of methylation of the population can be described by the temperature at which half the dsDNA molecules are melted and half are intact (*T50*). The higher the *T50*, the more the methylation in the DNA population. The normalised melt curve and the individual parameters derived from it can be simply computed using the raw fluorescence data exported from the real-time thermocyler or other microvolume fluorometer integrated with a rapid temperature cycler.

### Melt curve analysis of synthesised oligonucleotides

We tested the application of this analysis using synthesized oligonucleotides of known methylation. Three different oligonucleotides were synthesised, each representing a bisulfite modified DNA fragment of the same region of the CDKN2A promoter but differing in the extent of methylation, the number of methylated CpGs in the sequence. These CDKN2A oligonucleotide sequences are shown in Table 2. The region was 84 nucleotides long, and contained seven CpGs in total, six CpGs between the primer binding sites. In the oligonucleotide representing unmethylated bisulfite modified CDKN2A, the cytosines of the six CpGs were substituted with thymines. For the methylated oligonucleotide these cytosines remained as cytosines. For the partially methylated oligonucleotide three of the cytosines were substituted with thymines, the other three remained as cytosines.

**Figure 2:**
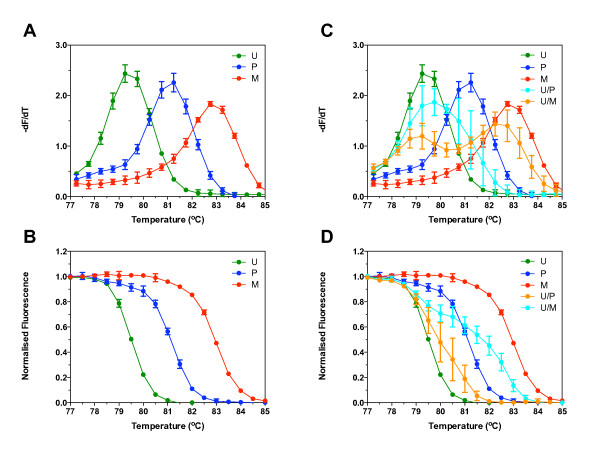
**Melt curves for CDKN2A oligonucleotides**. Oligonucleotide sequences representing a fragment of bisulfite modified unmethylated (U), partially methylated (P) or fully methylated (M) CDKN2A promoter (Table 2) were amplified using the CDKN2A primers and melted. The raw fluorescence data was converted to either the negative first derivative of the fluorescence with respect to temperature (-dF/dT) (A), or normalised (B), and plotted against temperature. Unmethylated oligonucleotide was spiked with either partially (U/P) or fully methylated (U/M) oligonucleotide, amplified using the CDKN2A primers and melted. Raw fluorescence data was either converted to -dF/dT (C), or normalised (D) and plotted against temperature. Data shown are the mean ± standard deviation of triplicate reactions.

Figure 2A shows the derivative peaks (-dF/dT), and Figure 2B the normalised melt curves, calculated as described, for the PCR products amplified from each of the three oligonucleotides. The general shapes of the -dF/dT plots and the normalised curves are similar, but they are hotter in proportion to the extent of methylation. The melt curve for the partially methylated oligonucleotide (three cytosines and three thymines) is between that of the unmethylated and methylated oligonucleotides. The *T50*, *Tto *and *Tdo *significantly increased with the extent of methylation (Table 3; P < 0.005 for all comparisons), demonstrating that differences could be measured between oligonucleotides which differed by as few as three methylated CpGs per molecule. The normalised melt curves did not differ whether the PCR products were melted immediately at the end of the PCR, or on a subsequent day, or had been melted a number of times before, even though the magnitude of the fluorescence intensity may have decreased over the time (data not shown).

**Table 3:** *T50*, *Tto*, *Ttd *and *Ttd – Tto *values for known oligonucleotides

Oligonucleotide	*T50*	*Tto*	*Ttd*	*Ttd – Tto*
Unmethylated	79.07 ± 0.06	78.48 ± 0.05	80.60 ± 0.06	2.12 ± 0.07
Partially methylated	80.00 ± 0.26	78.85 ± 0.53	82.39 ± 0.17	3.54 ± 0.47
Fully methylated	82.10 ± 0.00	81.13 ± 0.05	84.29 ± 0.03	3.16 ± 0.15
U/P	79.27 ± 0.21	78.30 ± 0.05	81.47 ± 0.18	3.17 ± 0.15
U/M	80.17 ± 0.15	78.45 ± 0.11	83.36 ± 0.19	4.90 ± 0.21

U/P – unmethylated oligonucleotide spiked with partially methylated oligonucleotide.

U/M – unmethylated oligonucleotide spiked with fully methylated oligonucleotide.

All values are the mean ± standard deviation of 3 replicate reactions.

Next, we investigated the ability of the method to analyse a mixture of two different oligonucleotides. Unmethylated oligonucleotide was spiked with either partially or fully methylated oligonucleotide, amplified and then melted. Spiking the unmethylated oligonucleotide with either partially methylated (U/P) or fully methylated (U/M) oligonucleotide significantly altered the shape of the -dF/dT (Figure 2C) and the normalised (Figure 2D) melt curves. The spiking did not significantly alter *Tto*, but did significantly increase *T50 *and *Ttd *in proportion to the extent of methylation of the spiked mixture (Table 3; P < 0.005 for all comparisons).

### Melt curve analysis of mixtures of methylated and unmethylated cell DNA

**Figure 3:**
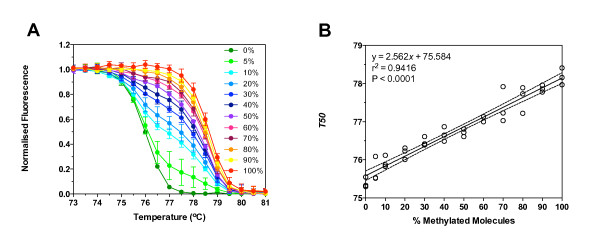
**Correlation between percentage of methylated reference and *T50***. Unmethylated reference (bisulfite modified normal donor lymphocyte DNA) and methylated reference (bisulfite modified CpG methylase treated normal donor lymphocyte DNA) were mixed so that the final percentage of methylated reference in the unmethylated reference was 0%, 5%, 10%, 20%, 30%, 40%, 50%, 60%, 70%, 80%, 90%, or 100%. The mixtures were amplified using the TIMP3 primers and melted. A) Graph of normalised fluorescence plotted against temperature. Data shown are the mean ± standard deviation of triplicate reactions. B) Linear correlation between percentage of methylated reference and *T50*.

To determine the relationship between *T50 *and the ratio of methylated to unmethylated molecules, we made dilutions of methylated reference DNA into unmethylated reference DNA. The dilutions were amplified using TIMP3 primers, the products melted, and the melt data normalised (Figure 3A). A visible shift to the right of the unmethylated reference was observed when 5% of the template molecules were methylated. There was a linear correlation between the percentage of methylated molecules in the mixture and the *T50 *(Figure 3B). Similar results were observed for regions of the CDKN2A and MGMT genes, except that shifts in the melt curves could be detected when 1% of the template molecules were methylated (data not shown).

### Melt curve analysis of cell line DNA

**Figure 4:**
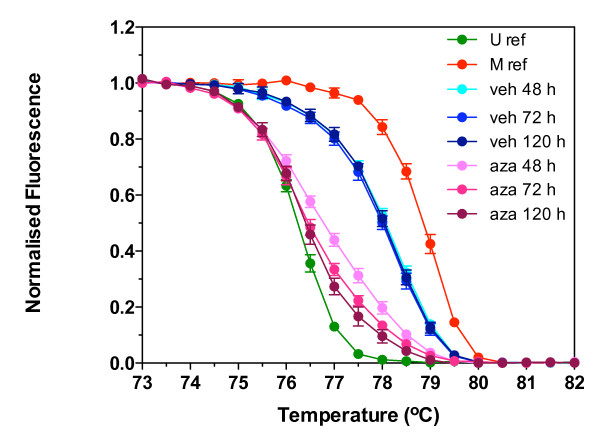
**Normalised melt curves of TIMP3 in OE33 cells treated with vehicle or 5-aza-2'-deoxycytidine**. Triplicate cultures of esophageal adenocarcinoma cell line OE33 were treated with either vehicle (veh) or 1 μM 5-aza-2'-deoxycytidine (aza) for 48, 72, or 120 h. Bisulfite modified DNA from treated OE33, unmethylated reference (U ref) and methylated reference (M ref) was amplified using the TIMP3 primers, melted, and the fluorescence normalised. Data shown for veh or aza treated OE33 are the mean ± standard deviation of triplicate reactions for each of triplicate cultures. Data shown for U ref and M ref are the mean ± standard deviation of triplicate reactions.

To validate melt curve analysis in cell line DNA we looked for a correlation between the melt curve parameters and the reduction in methylation in a cell line cultured with the demethylating agent 5-aza-2'-deoxycytidine (aza). We have previously shown that TIMP3 is methylated in the OE33 cell line, and that with aza treatment TIMP3 methylation is reduced and expression increased [14,15]. The results in Figure 4 show the normalised melt curves for DNA isolated from cells treated with vehicle or aza for 48, 72 or 120 h. Compared to unmethylated and methylated reference, all normalised melt curves for vehicle treated OE33 gave an intermediate melt, suggesting that OE33 was partially methylated. Incubating the cells with vehicle for increasing lengths of time did not alter the normalised melt curves, nor the *T50*, *Tto*, or *Ttd *(Table 4). Treatment with aza shifted the normalised melt curves to the left, with the degree of shift increasing with the length of treatment (Figure 4). The shifts in the normalised curves corresponded to a significant reduction in *T50*, *Tto *and *Ttd *when compared to vehicle. The *Tdo – Tto*, a measure of methylation heterogeneity, decreased with length of aza treatment (Table 4). Methylation was confirmed by COBRA [13]. Similar shifts in the normalised melt curves were observed for MGMT (data not shown).

**Table 4:** *T50*, *Tto*, *Ttd *and *Ttd – Tto *values for TIMP3 in OE33 cells treated with vehicle or 5-aza-2'-deoxycytidine

Sample	*T50*	*Tto*	*Ttd*	*Ttd – Tto*
U ref	75.52 ± 0.07	74.74 ± 0.16	77.36 ± 0.08	2.62 ± 0.24
M ref	78.07 ± 0.23	77.26 ± 0.37	79.83 ± 0.02	2.57 ± 0.35
veh 48 h	76.87 ± 0.18	75.63 ± 0.29	79.37 ± 0.04	3.74 ± 0.29
veh 72 h	76.80 ± 0.11	75.56 ± 0.17	79.33 ± 0.07	3.77 ± 0.19
veh 120 h	76.89 ± 0.18	75.70 ± 0.33	79.34 ± 0.08	3.65 ± 0.36
aza 48 h	75.40 ± 0.66	73.84 ± 1.15	78.90 ± 0.09	5.05 ± 1.12
aza 72 h	75.54 ± 0.42	74.34 ± 0.75	78.69 ± 0.06	4.35 ± 0.74
aza 120 h	75.49 ± 0.33	74.38 ± 0.57	78.45 ± 0.19	4.07 ± 0.64

U ref – unmethylated reference.

M ref – methylated reference.

veh – OE33 esophageal adenocarcinoma cell line treated with vehicle for 48, 72 or 120 h.

aza – OE33 esophageal adenocarcinoma cell line treated with 1 μM 5-aza-2'-deoxycytidine for 48, 72 or 120 h.

Data shown for veh or aza treated OE33 are the mean ± standard deviation of triplicate reactions for each of triplicate cultures. Data shown for U ref and M ref are the mean ± standard deviation of triplicate reactions.

### Melt curve analysis of tissue DNA

**Figure 5:**
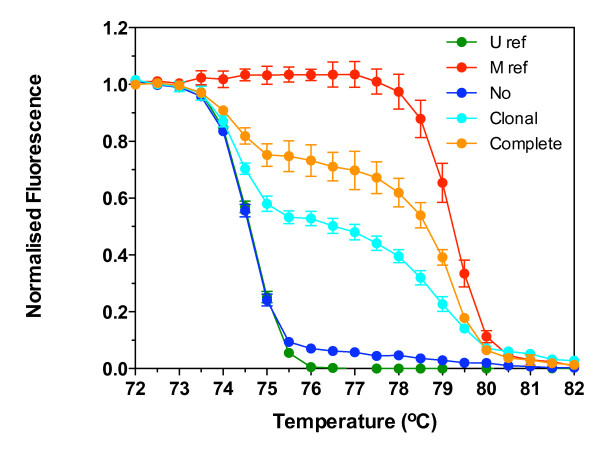
**Normalised melt curves of MGMT in formalin fixed paraffin embedded colorectal carcinoma tissues**. DNA was isolated from whole sections of FFPE CRC tissues that had either no, clonal, or complete loss of MGMT expression as demonstrated by immunohistochemistry. Bisulfite modified DNA from CRC tissues, unmethylated reference (U ref) and methylated reference (M ref) was amplified using MGMT primers, melted and the fluorescence normalised. Data shown are the mean ± standard deviation of six replicate reactions.

To demonstrate the utility of our melt analysis for detecting different patterns of methylation in tissue samples, methylation was measured in bisulfite modified DNA prepared from whole sections of FFPE CRC tissue with variable expression of MGMT as demonstrated by immunohistochemistry. Methylation of MGMT is associated with a reduction of its mRNA and protein expression in cancer cell lines and tissues [15,17]. Sections were taken from a single block of tissue that had either no, clonal, or complete loss of MGMT expression in the tumor cells. The normalised melt curves for a region of the MGMT promoter are shown in Figure 5. The CRC with no detectable loss of MGMT expression by immunohistochemistry melted at the lowest temperature, that with complete loss the highest temperature, and that with clonal loss melted at an intermediate temperature. The *T50*, *Tto*, *Ttd *and *Ttd – Tto *are presented in Table 5. The *T50 *significantly increased with increasing loss of expression. There was no significant difference in the *Tto *for unmethylated reference and any CRC tissue. The *Tto *for the unmethylated reference and each of the CRC tissues were significantly less than the methylated reference, indicating the presence of unmethylated molecules in each of the CRC tissues. This would be expected due to the presence of stromal and other non-tumor cells in the tissue section. The *Ttd *for the methylated reference and each of the CRC tissues was significantly more than the unmethylated reference, indicating the presence of methylated molecules in all the CRC tissues, including the tissue with no detectable loss of MGMT protein expression. The patterns of methylation were confirmed by direct bisulfite sequencing (Additional File 1).

**Table 5:** *T50*, *Tto*, *Ttd *and *Ttd – Tto *values for MGMT in FFPE CRC tissues

Sample	*T50*	*Tto*	*Ttd*	*Ttd – Tto*
U ref	74.17 ± 0.04	73.64 ± 0.04	75.54 ± 0.04	1.90 ± 0.03
M ref	78.70 ± 0.25	78.06 ± 0.37	80.43 ± 0.15	2.36 ± 0.25
No	74.18 ± 0.09	73.54 ± 0.06	77.24 ± 1.01	3.71 ± 0.95
Clonal	75.84 ± 0.21	73.62 ± 0.17	81.12 ± 0.37	7.50 ± 0.31
Complete	76.17 ± 0.07	73.70 ± 0.09	80.32 ± 0.45	6.62 ± 0.40

U ref – unmethylated reference.

M ref – methylated reference.

No, Clonal and Complete – whole sections of FFPE CRC tissue with either no, clonal or complete loss of MGMT protein expression in tumor cells, as determined by immunohistochemistry.

All values are the mean ± standard deviation of 6 replicate reactions.

## Discussion

We describe a simple method to quantitate DNA methylation using the raw fluorescence melt data obtainable following PCR amplification of bisulfite modified DNA. The method, which provides a summative measure of methylation at all the CpGs in the PCR product, is reproducible, sensitive, and informative whether the methylation in the region amplified is homogeneous or heterogeneous. While the differences due to methylation were apparent visually, key descriptive parameters (*T50*, *Tto, Ttd and Ttd – Tto*) could be calculated mathematically, which permits statistical analysis of results and eliminates the subjectivity of visual assessments. We validated the utility of the method with synthesised oligonucleotides and DNA prepared from fresh cells and formalin fixed paraffin embedded tissues.

In the PCR of bisulfite modified DNA, what were unmethylated cytosines in the genomic DNA will be amplified as thymines, and only methylated cytosines will be amplified as cytosines. These sequence differences can be distinguished by the melt curves of the amplified products. The temperature range over which melting occurs and the shape of the curve is a function of the length, sequence and GC content of the product. Products derived from DNA template containing methylated cytosines will have a higher GC content, and so a higher melt temperature and touch down temperature, than otherwise equivalent products from DNA with unmethylated cytosines. Similarly, products from unmethylated DNA will have a cooler melt temperature and lower take off temperature than otherwise equivalent methylated DNA. The difference between the take off and touch down temperatures will reflect the heterogeneity (or uniformity) of methylation in the template molecules. Melt curve analysis, unlike gel electrophoresis, can distinguish between products which are of the same length but have different GC/AT ratios.

Several groups have used the melt curves from PCR products to detect mutations, polymorphisms or methylation. Worm [9], Akey [18] and Guldberg [19] detected methylation by comparing the melting temperature (*T*m) from the derivative peaks (-dF/dT) of the melt curves of the PCR products. While populations containing only methylated or unmethylated molecules are easily distinguishable by the difference in *T*m, heterogeneous mixtures of unmethylated, partially methylated and fully methylated molecules are difficult to analyse or compare. Lorente [12] described a technique called MCA-Meth in which they calculated a ratio of the relative height of the -dF/dT peaks for unmethylated to methylated molecules. The MCA-Meth is semi-quantitative, and cannot be used if partially methylated molecules are present.

Wittwer described a normalisation of the raw data from high resolution melts made possible with the development of the Idaho HR-1 instrument for genotyping and mutation [8]. To derive the normalised curves the experimenter had to select the 100% and 0% fluorescence values by eye. Sequence alterations, such as due to methylation, were identified visually by changes in the shape or position of the normalised curve. Wojdacz [10] used high resolution melting to measure methylation following amplification in the Rotor-Gene 6000 with SYTO9 dye. Mixtures of fully methylated or fully unmethylated DNA molecules, but not partially methylated molecules, were used in the validation of their method. In the analysis the raw melt curves were adjusted, in a manner not described, so that all the samples had the same starting and ending fluorescent signal. In these high resolution melt methods methylation differences between samples was assessed subjectively, not quantitatively. To the best of our knowledge, we are the first to describe and validate a method to quantitate DNA methylation from melt curves that can be used for samples that contain any mixture of methylated, unmethylated and/or partially methylated molecules, and does not depend on visual analysis.

Our method is based on normalising the raw melt fluorescence data using a simple algorithm which is easily computerised. It is easy to assess methylation visually from the normalised melt curve, but the power of the method is that it permits a mathematical analysis of the methylation. From the normalised melt data we calculate four parameters which describe the methylation status of the amplified region. The *Tto*, the temperature at which melting begins, reflects the least methylated amplicons present. The *Ttd*, the temperature at which the product is completely melted, reflects the most methylated amplicons present. The *Ttd – Tto *reflects the heterogeneity of the amplicons with respect to methylation. If *Ttd – Tto *is small, most alleles have a similar amount of methylation, if it is large then some alleles are high in methylation, others low. The *T50 *is the temperature at which half of the amplicons are melted, and reflects the sum of all methylation of all the CpGs in the amplified region. The more the methylation in a region, the higher the *T50*. Because these parameters are derived mathematically, the subjectivity of other melt curve analytical methods is eliminated.

To validate our method we first used synthesised oligonucleotides representing a bisulfite modified sequence from the CDKN2A promoter in its methylated, unmethylated and partially methylated form to show that the shape of the normalised melt curve, and the temperature at which the melting commenced and was completed, reflected the degree of methylation of the template alleles. We then demonstrated the utility of the method in more the complex situation of analysing DNA preparations from fresh cells and formalin fixed paraffin embedded tissues. Using lymphocyte DNA methylated with CpG methylase diluted into unmethylated lymphocyte DNA we showed a linear relationship between *T50 *and the percentage of methylated amplicons in the mixture. The method could unambiguously detect methylation in samples containing between 1–5% of methylated DNA, depending on the target sequence. We were able to quantitate the anticipated reduction in methylation (*T50*) and methylation heterogeneity (*Ttd – Tto*) over time in a cell line treated with the demethylating agent 5-aza-2'-deoxycytidine. Finally, we could quantitate differences in methylation associated with complete, clonal or no loss of expression of MGMT expression in formalin fixed paraffin embedded tissues. Our melt analysis of methylation was confirmed by bisulfite sequencing.

The melt curve analysis is a valuable addition to the methods available for measuring methylation. The most common method for the analysis of methylation is methylation specific PCR (MSP), which uses primers that are specific for either methylated, or unmethylated, bisulfite modified DNA. This technique relies on 3' mismatching of the PCR primers for specificity. False positives can occur if the primers are poorly designed or the PCR is run at too low a temperature, or possibly for too many cycles. The method is sensitive, but only measures methylation of one or two CpGs located near the 3' end of the primers and is not quantitative. Tumors can be classified as methylated when only a minor percentage of cells are methylated, or if the bisulfite conversion of the DNA is incomplete (with some unmethylated cytosines remaining as cytosines, not being converted to uracils). Pyrosequencing accurately quantifies DNA methylation levels for multiple CpG sites within the PCR product, but it requires more expensive, biotin labeled primers, and a pyrosequencer in addition to a PCR thermocycler. MethyLight is an extremely sensitive and quantitative assay which uses TaqMan technology to measure methylation, utilising the cleavage of a dual-labeled fluorogenic hybridization probe by the 5' nuclease activity of Taq polymerase during the PCR amplification. The probes are expensive, and are specific for a particular methylation pattern within the amplified region. Generally the probes are designed to detect the fully methylated or fully unmethylated allele only, not partially methylated alleles. Bisulfite sequencing is the gold standard for methylation analysis, but is time consuming and expensive, and its accuracy is limited by the number of clones which are sequenced. Melt curve analysis is rapid and cost-effective method to quantitate DNA methylation when information about the summative methylation of the amplified region is required. Unlike MSP, it is quantitative and does not generate false positives. Unlike pyrosequencing it does not require equipment other than a PCR thermocycler, and unlike MethyLight it resolves heterogeneous methylation and can quantitate mixtures of variably methylated molecules in the same reaction, and does not require specific probes. It is much quicker than bisulfite sequencing, but does not provide the same detail about the pattern of methylation. It is particularly suited to measuring methylation in CpG rich regions, such as CpG islands associated with the promoter regions of genes, where it is generally sufficient to measure average CpG methylation levels rather than the level for every single CpG [20]. If high resolution methylation detail is required, such as can be provided by bisulfite sequencing, melt curve analysis can be used to screen for samples or clones which are unmethylated and do not require further analysis.

Two modifications have the potential to improve the performance of the method, although the principles of the analysis would not change. Fluorescent dyes such as SYTO9, LC Green or Eva Green which do not redistribute during melting may improve the detection of minor unmethylated populations, increasing sensitivity. Instruments which acquire fluorescence data with greater accuracy and over smaller temperature increments (e.g., 0.01°C compared to 0.5°C with the RG3000 used in this study) would also be expected to improve the sensitivity of the assay.

## Conclusion

We have developed a rapid, reproducible and cost-effective in-tube assay to quantitate DNA methylation which yields significant information about the methylation in the template DNA. It resolves heterogeneous methylation, quantitating the total methylation when there are mixtures of fully methylated, partially methylated and unmethylated molecules in the same reaction, and requires no extra experimental processing after the PCR reaction. The primers are as easy to design as for a conventional PCR reaction, and expensive labelled probes are not required. The mathematical analysis does not rely on proprietary algorithms or software, is simple to computerise, and eliminates the imprecision in methods which require visual manipulation and interpretation of melt curves. The method is sensitive and reproducible for measuring methylation using SYBR Green I in a commonly used real-time thermocycler, and so is well suited to most research or routine laboratories.

## Competing interests

The authors declare that they have no competing interests.

## Authors' contributions

ES undertook the laboratory component of the study and participated in the design of the study, the analysis of the results, and the drafting the manuscript. MEJ participated in the design of the study and wrote the software for the melt analysis. PAD participated in the design of the study, the analysis of the results and the drafting of the manuscript. Each author read and approved the final manuscript.

## Pre-publication history

The pre-publication history for this paper can be accessed here:

http://www.biomedcentral.com/1471-2407/9/123/prepub

## Supplementary Material

Additional file 1 **Direct bisulfite sequencing of MGMT PCR product**. Following amplification and melting, the MGMT PCR products of the CRC tissues with no, clonal or complete loss of expression, unmethylated reference (U ref) and methylated reference (M ref) were electrophoresed on 2% agarose gels. The presence of a single product and its size were confirmed by staining the gel with ethidium bromide. Each of the bands from the six replicate reactions for each sample were excised from the gel, the replicate bands combined, and purified using QIAquick Gel Extraction Kit (Qiagen), following the manufacturers instructions. The purified products (1 – 4 ng) were sequenced using BigDye Terminators v 3.1 (Applied Biosystems Inc, Foster City, CA) and both forward or reverse MGMT primers. Click here for file
