# Supplementary figures and images for: Quantitation of DNA methylation by melt curve analysis
Source: BMC Cancer. 2009 Apr 24;9:123. doi: 10.1186/1471-2407-9-123 (PMC2679043; doi:10.1186/1471-2407-9-123)

**U ref**

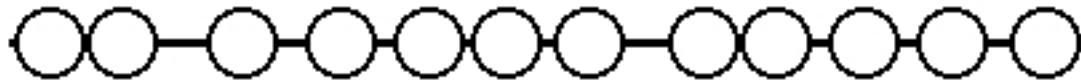

**M ref**

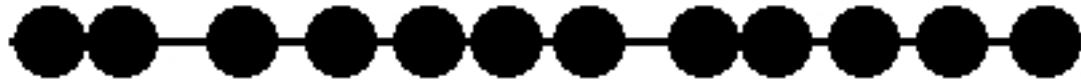

**No Loss**

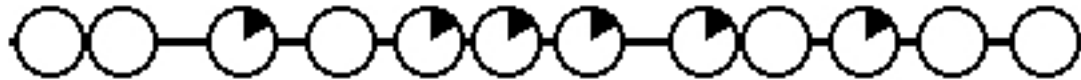

**Clonal Loss**

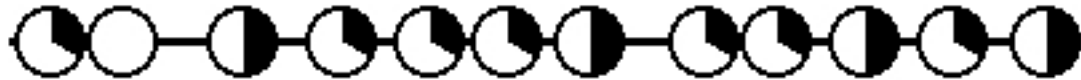

**Complete Loss**

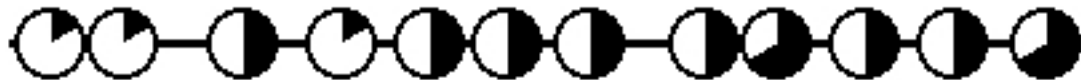

Supplement: Additional file 1 — Direct bisulfite sequencing of MGMT PCR product. Following amplification and melting, the MGMT PCR products of the CRC tissues with no, clonal or complete loss of expression, unmethylated reference (U ref) and methylated reference (M ref) were electrophoresed on 2% agarose gels. The presence of a single product and its size were confirmed by staining the gel with ethidium bromide. Each of the bands from the six replicate reactions for each sample were excised from the gel, the replicate bands combined, and purified using QIAquick Gel Extraction Kit (Qiagen), following the manufacturers instructions. The purified products (1 – 4 ng) were sequenced using BigDye Terminators v 3.1 (Applied Biosystems Inc, Foster City, CA) and both forward or reverse MGMT primers. [file 1471-2407-9-123-S1.pdf]
